# The Practices of Portuguese Primary Health Care Professionals in Palliative Care Access and Referral: A Focus Group Study

**DOI:** 10.3390/healthcare13131576

**Published:** 2025-07-01

**Authors:** Camila Barreto, Marcelle Miranda da Silva, Ana Fátima Carvalho Fernandes, Romel Jonathan Velasco Yanez, Carlos Laranjeira

**Affiliations:** 1 USF Buarcos, Local Health Unit of Baixo Mondego, Av. Dr. Mário Soares 64, 3080-254 Figueira da Foz, Portugal; camilajbarreto17@gmail.com; 2 School of Health Sciences, Polytechnic University of Leiria, Campus 2, Morro do Lena, Alto do Vieiro, Apartado 4137, 2411-901 Leiria, Portugal; 3 Escola de Enfermagem Anna Nery (EEAN), Universidade Federal do Rio de Janeiro (UFRJ), Afonso Cavalcanti Street, Rio de Janeiro 21211-110, Brazil; marcellemsufrj@gmail.com; 4 Department of Nursing, Federal University of Ceará, Fortaleza 60430-160, Brazil; afcana@ufc.br (A.F.C.F.); romebarce_95@hotmail.com (R.J.V.Y.); 5 Centre for Innovative Care and Health Technology (ciTechCare), Polytechnic University of Leiria, Campus 5, Rua das Olhalvas, 2414-016 Leiria, Portugal; 6 Comprehensive Health Research Centre (CHRC), University of Évora, 7000-801 Évora, Portugal

**Keywords:** palliative care, primary health care, professionals, access, referral, qualitative study, Portugal

## Abstract

**Background/Objectives**: The prevalence of people with incurable and progressive diseases in primary health care is high. Family doctors and nurses must be active agents in the early identification of palliative needs and the implementation of palliative approaches in cases of low to intermediate complexity. While there is a need for early referral of more complex palliative care (PC) cases to specialized teams, primary health care (PHC) professionals lack the confidence or skill to describe their role. This study sought to explore and describe (a) the practices of PHC professionals regarding their PC provision; (b) the barriers regarding access and referral of patients to specialized PC services; and (c) the strategies used or recommended to mitigate difficulties in accessing and referring to specialized PC. **Methods**: A descriptive qualitative study was carried out, using five focus groups conducted with nursing and medical staff at three local health units in the central region of Portugal. Semi-structured interviews were conducted, and then recorded, transcribed, and analyzed through a thematic analysis approach. The reporting of this research follows the COREQ checklist. **Results**: In total, 34 PHC professionals participated in this study. The majority of participants were women (n = 26) and family doctors (n = 24). Their mean age was 43.8 ± 11.9 (range: 29 to 65 years). The findings were organized into three core themes: (1) the contours of palliative action developed by PHC teams; (2) barriers to access and safe transition between PHC and specialized PC; and (3) ways to mitigate difficulties in accessing and referring to specialized PC. **Conclusions**: Our findings highlight the fundamental role of PHC professionals in providing primary PC, and in identifying PC needs and referring patients to PC early on, while exposing the systemic and interpersonal challenges that hinder these processes. To overcome these challenges, it is essential to invest in the development of integrated care models that promote practical, low-bureaucratic referral processes and capture the human resources necessary for the adequate follow-up of users.

## 1. Introduction

Over the last few decades, exponential population aging has been a trend observed worldwide, and the number of deaths occurring at older ages is expected to continue to increase [1,2]. This reality is especially notable in developed and developing countries, and also applies to the Portuguese population [3,4,5,6]. In parallel, today’s societies exhibit a high prevalence and incidence of chronic, incurable, and progressive diseases [2,7]. The rising prevalence of multimorbidities exerts escalating pressure on health and social institutions, which encounter difficulties in accommodating the evolving requirements of shifting populations and necessitate unique health considerations, especially toward the end-of-life (EoL) [1,2,7]

This scenario requires a palliative care (PC) approach to enhance patients’ quality of life, resulting in a heightened need and demand for such care [7,8,9,10]. Palliative interventions and early referral practices are known to reduce costs, improve the care experiences of people in PC and their caregivers, enable patients to make choices about their EoL care, and in some cases, increase life expectancy [9,11,12,13]. In particular, home-based PC has demonstrated significant benefits in meeting the needs of people with advanced diseases, in addition to reducing hospital admissions and emergency department visits, thus emphasizing person-centered care and reinforcing the sustainability of the health system [14,15].

The care model, composed of specialized community palliative care teams (CPCT), has been shown to improve the experience of EoL care, namely, by reducing the use of emergency services by around 50% [16]. In addition, it offers patients the possibility of dying at home: results found that 39% of patients with CPCT died in a hospital compared to 74.8% without CPCT [15,16]. In fact, the home is the preferred location for EoL care, both for patients and their families, with preferences varying between 11% and 89%. The home is also the preferred place of death, with preferences varying between 51% and 55% [17].

The World Health Organization estimated that, overall, only 14% of people who need PC currently receive it [18]. PC delivery differs significantly across countries, health systems, and communities. PC access is widely available in most high-income countries, where there has been a steady growth in the availability of specialized PC services, but it remains limited in low- and middle-income countries [19,20]. Even so, while the availability of specialized services has grown across Europe, the ratios per 100,000 people remain below the recommendations set by the European Association for Palliative Care (EAPC) [21]. In the Portuguese context, according to the 2023 Autumn Report of the Portuguese Palliative Care Observatory, universal coverage of PC resources in the country is far from being achieved. Data also reveal profound asymmetries among districts/regions and typologies. According to the EAPC recommendations and across the different types, Portugal requires an estimated total of 793 beds for PC at the national level (90 beds per million inhabitants). However, there are presently only 423 beds, which represents a national coverage rate of 53% (with 72% in acute care and 45% in non-acute care). Regarding CPCTs, the coverage rate at the population/structural level is 46% [22]. Clearly, in Portugal, access to PC still falls short of patient needs [22,23].

Given the increasing palliative needs and the deficient coverage of specialized PC, the teaching and training of skills for primary health care (PHC) professionals is necessary for earlier, more accessible, and global PC [7,11]. In this sense, family physicians and nurses must be active agents in the early identification of palliative needs and in the implementation of palliative approaches in cases of low to intermediate complexity [7,24], as they are often the first point of contact between patients and health care [19,25,26]. In more complex cases, they play an important role in early referral to specialized PC teams [24].

PHC covers most of an individual’s health needs throughout their lifespan, including prevention, treatment, rehabilitation, and EoL care [25,26,27]. It provides comprehensive care in the community, allowing access to care and dealing with all health problems (regardless of age, sex, or any other characteristic of the person in question) [25,26,28], from the individual’s conception until their death (and extending into the mourning period) [19,24,25]. Furthermore, the proximity of family doctors and nurses to users and families favors the therapeutic relationship, and the involvement of these teams in PC is associated with greater patient satisfaction [29]. The skills and characteristics of PHC teams are based on principles and objectives that are common to the PC paradigm. Both seek health care that is centered on the person (and not just the disease), with continuous care, with a global and holistic vision, and both highlight the importance of addressing physical, psychological, social, and spiritual issues of the ill person and their family unit [25,26,27,30].

Critically, PHC professionals need to understand how they can provide PC in their context, and when they should refer to specialized PC, within a collaborative and integrated network [10,24,28,31]. Although studies highlight the challenges in referring and providing PC by PHC professionals [19,30,32,33], there are no known studies that focus on the Portuguese reality, justifying the present study. Thus, we intend to explore the following: a) the practices of PHC professionals regarding their PC provision; b) barriers regarding access and referral of patients to specialized PCs; and c) the strategies used or recommended to mitigate difficulties in accessing and referring to specialized PCs. In this way, we hope to contribute to a better understanding of the articulation of PHC and PC in the Portuguese reality to understand what difficulties professionals face and how clinical practice could be improved.

## 2. Materials and Methods

### 2.1. Study Design

This is a qualitative, exploratory, and descriptive study using focus groups (FGs), following the proposal of Krueger and Casey [34]. The study is anchored in a subjective view of the subject and linked to a constructivist perspective of reality, exploring perceptions in a categorized way and integrating their theoretical references [35]. Qualitative research is an evolutionary and adaptive approach that focuses on people’s experiences, behaviors, and opinions. It seeks to answer “why” and “how” questions, providing detailed insight and understanding that quantitative methods cannot achieve [36,37].

Focus groups (FGs), in particular, are a form of interview designed to gather multiple perceptions on a particular topic/area of interest and are an increasingly used qualitative research method [38,39]. Unlike individual interviews, FGs provide the additional dimension of interactions between group members [40]. In the context of health care and medical research, FGs are particularly relevant, as they suggest explanations for behaviors that could have a sociocultural context [40].

The study followed the consolidated criteria for reporting qualitative research (COREQ) checklist [41] (Supplementary File S1).

### 2.2. Setting and Participants

Five FGs were organized with primary health care doctors and nurses from three local health units (LHUs) in the central region of Portugal. Each FG was composed of professionals assigned to the same LHU, with a number varying between six and eight participants (out of a total of 34). For the recruitment process, a purposive sampling strategy was used [42], and the following inclusion criteria were established: (1) doctors and nurses with at least one year of experience in PHC; (2) professionals who provide direct care to patients and their families, particularly to people who routinely required referral to PC. Exclusion criteria: professionals absent from work at the time of data collection (due to illness, training, or vacation). The application of eligibility criteria was assessed by the principal investigator. The data saturation criterion was used to determine the number of participants [43]. Recruitment ceased when data no longer provided meaningful information [44]. To obtain maximum sample variation, no restrictions were imposed on sex, age, and professional seniority.

### 2.3. Data Collection

Data were collected using semi-structured interviews, between October and November 2024, during working hours to facilitate attendance. FGs were moderated by an experienced female doctor (C.B.) and assisted by a faculty researcher (C.L.) and had an average duration of 75 min (range: 30 to 100 min). The participants were unfamiliar with the researchers before data collection. Data were collected in each clinical site, and the choice of location for the in-person interviews followed FG recommendations [34] in terms of accessibility, comfort for participants, as well as the confidentiality of the information shared.

Potential participants were contacted by the principal investigator via email, who provided details about the study and FG scheduling, including time and location. Consent was obtained from contacts who were willing to participate in the study. Each participant completed a sociodemographic and professional characterization with questions about their age, gender, professional category, qualifications, years of professional experience in primary health care, years of overall professional experience, training in PC, and self-assessment of the level of perceived competence for PC practice (on a scale of 0 = no level to 10 = high level).

A guiding script was followed for each FG session, consisting of four distinct parts: introduction, when the main researcher/moderator thanked the group for their availability and participation and presented the study’s components; FG legitimacy, obtaining authorization to record the audio of the session, providing the informed consent form and ensuring the confidentiality of the information; development, presenting the purpose and objectives of the study, and posing questions to encourage discussion and group discussion; and finally the conclusion, when the researcher thanked everyone for their availability and collaboration, made themself available for future clarifications and guaranteed that the findings would be available for consultation [34].

A pilot FG discussion was performed to evaluate the interview guide questions and to facilitate effective collaboration between the moderator and the observer. The final study incorporated data from the pilot FG, as only minimal modifications were applied to the interview guide.

The main researcher/moderator encouraged participants to openly share their experiences and stressed the importance of confidentiality of observations made during discussions and tolerance of different perspectives. The discussion in each FG was initiated using the presentation of a clinical vignette, which consisted of a clinical case of a user with several palliative needs, requiring a reflective analysis by the participants. Participants are more willing to share their views when using the vignette approach as it allows some distance between their own experience and the given scenario [34]. This vignette served as an “icebreaker”, followed by the question, “What palliative needs do you identify in this clinical case?” Thereafter, the moderator introduced open questions from the interview script, guided by the literature and primary study objectives, and refined by discussions among the authors. Questions covered the following topics: (1) intervention practices in PC; (2) access and referral to specialized PC; (3) facilitating factors and barriers; and (4) strategies used or to be implemented for referral [7,45,46,47]. Encouraging affirmations and direct inquiries were employed to facilitate interaction and stimulate additional debate on these subjects of interest. The moderator’s assistant noted privileged information about facial expressions, gestures, tone of voice, and the context in which the discourse was delivered. These aspects are fundamental in the process of decoding, interpreting, and analyzing data. No repeat interviews were carried out. Participants were neither provided with the transcripts for feedback or changes nor solicited for commentary on the outcomes.

### 2.4. Data Analysis

**Figure 1:**
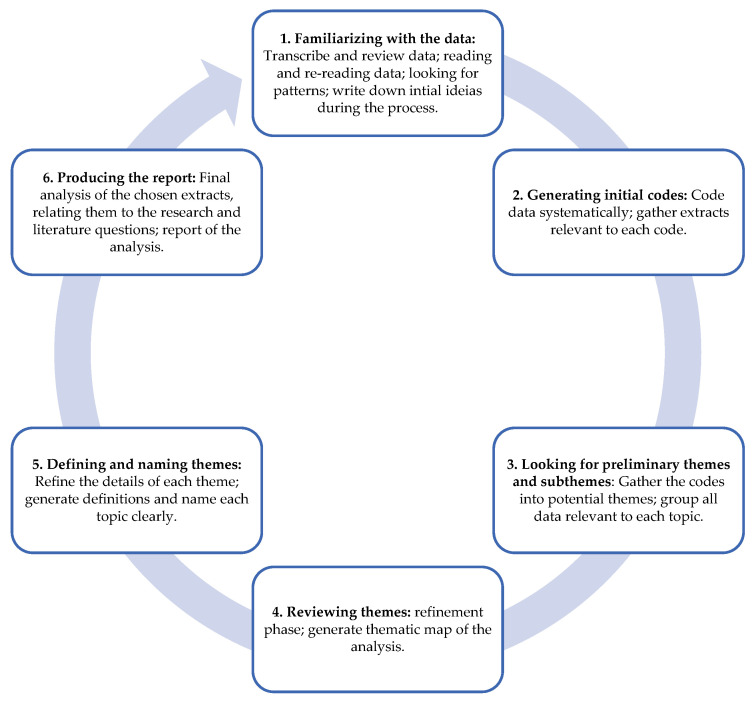
Six-step Braun and Clarke [48,49] thematic analysis.

Thematic analysis of the data was performed according to the proposal by Braun and Clarke [48,49] (Figure 1).

The interviews were audio-recorded and manually transcribed to maintain the authenticity of the original meanings. The first and last authors thoroughly analyzed the transcripts and familiarized themselves with the data, including the context in which extracts were produced; thus, the interpretation was supported by a systematic analysis of the data. Preliminary codes were categorized into themes and refined following each interview. An endeavor was undertaken to transition from descriptive to interpretative codes to discern wider links among participant experiences. After each interview, the coding matrix was rigorously analyzed to identify the most pertinent elements of participant experiences, culminating in an agreement among researchers through discussion [44].

The interviews were loaded into the qualitative data analysis program WebQDA (Version 3.0, University of Aveiro, Aveiro, Portugal) for data storage and administration. After the analytical procedure, the textual excerpts, initially in European Portuguese, were translated and subsequently “back-translated” to ensure the retention of their original meaning.

### 2.5. Trustworthiness

The Guba and Lincoln criteria were used to maintain study rigor [50]. To ensure credibility, we used quotes from participants that faithfully expressed their narrative. Qualitative data from other sources, such as transcripts and researcher notes, were also used. Triangulation of researchers was used to refine the findings and respective thematic analysis. To ensure transferability, the study design, participants, context, sampling methods, data collection, and analysis were described in detail. To ensure reliability, the work was subjected to an external audit by independent researchers. Finally, confirmability was achieved by maintaining reflective reporting throughout the data collection and analysis phases. Through peer debriefing and reflective journaling, biases and reflections were documented, increasing transparency and minimizing subjectivity.

The research team used a hybrid approach (i.e., including both deductive and inductive coding strategies) to form thematic categories [48,49]. Several research meetings were conducted to facilitate the sharing and comparison of findings, helping to identify recurring themes. When differing opinions arose, a consensus was reached through discussion. The main researcher is a resident physician in general and family medicine, with clinical experience with chronic and palliative patients, and is undergoing postgraduate training in PC. The fourth author (R.J.V.Y.) is a registered nurse with experience in PHC and PC. The remaining authors (M.M.S., A.F.C.F., and C.L.) hold PhD degrees and are faculty members with expertise in using qualitative research. Before collecting and analyzing the data, each author documented and acknowledged their preconceptions, thus allowing their conscious use, particularly in the development of the interview guide, and thereby fostering enhanced and/or novel insights into the subject of study.

### 2.6. Ethical Considerations

The present study was carried out in accordance with the principles of the Declaration of Helsinki and after the favorable opinion of the Local Ethics Committee (CE/IPLEIRIA/87/2024). Participation was voluntary and was accompanied by the completion of the informed consent form. The study’s goal was clearly articulated to all participants, and confidentiality of their identity and reports was guaranteed. The interview recordings were stored in a password-protected file accessible solely to the researchers and will be deleted one year after the project’s conclusion. The lead researcher informed participants that they could interrupt the interview at any time without any associated consequences. Participants received no benefit and incurred no risks of harm or costs for their participation in this study. Alphanumeric codes (FG1, FG2, …) were used, thus ensuring transparency in the documentation of observations and expressing consensus among participants.

## 3. Results

### 3.1. Sample Description

In total, 34 primary health care professionals participated in this study. The majority of the participants were women (n = 26), and the mean age was 43.8 ± 11.9 (range: 29 to 65 years). Regarding the professional category, most participants were doctors (n = 24), and the rest were nurses (n = 10). Most participants had no training in PC (n = 23). Notably, the average level of self-perceived competence in PC was low (3.9 ± 1.7). A sample background is provided in Table 1.

### 3.2. Findings Overview

The findings were articulated through three themes and ten subthemes (Table 2) that bring together the key elements to respond to the study’s objectives. Initially, the practices of primary health care professionals regarding their provision of PC were explored. Subsequently, how professionals ensure access and referral of patients and families to specialist PC was described. This exploration resulted in the identification of barriers that participants face in this process, as well as the strategies they use or would recommend to mitigate the difficulties experienced.

#### 3.2.1. Theme 1—The Contours of Palliative Action Developed by PHC Teams

A consensus developed among FGs that PC targets individuals with incurable diseases, characterized by a limited life expectancy, where alleviation of suffering is paramount to the therapeutic approach. The participants indicated that palliative principles were beneficial, regardless of diagnosis, whether the patient had slowly progressing dementia or an advanced cancer. This theme includes three subthemes: (a) identification of palliative needs; (b) the primacy of the therapeutic relationship; and (c) limitations in response capacity.

**(a)**  **Identification of palliative needs**

Participants described ease in identifying and assessing palliative needs through the use of decision-making support tools. However, most participants revealed ambivalence in the process of assessing non-oncological palliative needs, particularly in patients with dementia, heart failure, or chronic respiratory diseases.

*FG1: When assessing palliative needs, sometimes the difficulty is not so much with cancer, but with non-oncological diseases, namely heart failure, COPD, dementia, which could benefit from some (palliative) monitoring. We have some difficulty understanding these needs.*
*FG2: The first step is to demystify the idea that palliative care is only for oncological diseases, which is what we hear about most. There is no doubt that for these conditions we are much more aware to identify palliative needs.*
*FG4: Oncological situations are easy to identify. Perhaps, if we think more about chronic diseases, for example heart failure with decompensation, then it might be more difficult. These are situations that people don’t associate with palliative care.*
*FG5: The use of instruments such as the Edmonton Symptom Assessment, the Performance Palliative Scale, or the surprise question are important resources for assessing needs.*

Furthermore, participants highlighted that, in cases of chronic non-oncological diseases, the response often underestimates the severity of the situation, due to the long course of the disease and the aging process.

*FG4: That’s definitely more difficult, because we end up devaluing it. As a rule, they are also older people. It is much easier to assess an oncological situation than a non-oncological chronic disease.*

**(b)**  **The primacy of the therapeutic relationship**

Another aspect that was consensual among the FGs is related to the proximity of primary health care professionals to patients and families. Being a PHC professional means having the opportunity to accompany different generations of the same family throughout their life cycle, allowing one to build lasting therapeutic relationships that impact the quality of care offered.

*FG1: The fact that we follow people throughout their lives makes it easier for us to reach the person. Many times, patients accept it better when we tell them, even if we don’t have to be the ones to do it, even if the diagnosis was provided elsewhere. Also, because we don’t really know what has been said or not… Other times they come to clarify things that the hospital doctor didn’t convey, or that they didn’t hear or didn’t understand.*
*FG4: It’s giving support. It’s knowing how to listen. It’s giving that hug. The user may be trying to deal with the situation alone, but then their daughter, their wife comes to us to support them in the background.*
*FG2: Often, being family doctors and nurses can be an advantage, because we know the person better.*

Communication is an important catalyzer in promoting a person’s self-determination. Participants highlighted the need for active listening and information management about what the person wants to know about their illness or what they intend to share about the illness with their family support network.

*FG5: There are many people who prefer not to know, and this is not always respected (…). We must always ask what the person knows and what they want to know. We have to give the patient space, they may not want the family to get involved, so the family doesn’t suffer. We must demystify this with them, but at the same time respect their decision.*

**(c)**  **Limitations in response capacity**

The professionals mentioned several situations in which they provided general PC, with emphasis on symptomatic control in the initial phases, referral and mobilization of resources/support in the community, as well as involvement of families.

*FG1: With our approach we achieve initial pain control, but in more advanced situations this no longer happens. Also for constipation and nausea, if applicable. We can answer simpler questions, even if they are not in situations of terminal illness, but in which the principles of palliative care apply.*
*FG2: We can manage pain and some symptoms in the earliest stages. In social terms, users often have doubts, particularly about the support and benefits they may be entitled to. As we are not social workers and do not know how to clarify all doubts, we refer the person to the Social Worker and make ourselves available for whatever is necessary.*
*FG3: We adjust medication, mobilize some social resources and provide support to the family.*
*FG4: We can and should provide palliative care! It is the only way to give access to the population (…). We monitor low complexity situations or pass this responsibility on to specialized care in moderate to high complexity situations given our limitations.*

Despite existing efforts, there is a feeling that little is being done to help people die at home (the location preferred by most patients).

*FG5: Despite everything, we do very little. Most of our users die in the hospital. If we don’t issue death certificates, it’s because they die in hospital… the truth is that we are doing little, especially in this matter of allowing people to do this journey at home, which we know will not be for everyone, but perhaps the vast majority would be able to do so (…).*

#### 3.2.2. Theme 2—Barriers to Access and Safe Transition Between PHC and Specialized PC

Primary health care professionals face several barriers when it comes to the transition of care and the referral process. Four subthemes were identified: (a) myths and misconceptions regarding PC; (b) early referral conditioned by the heterogeneity of disease trajectories; (c) bureaucratization of processes and difficulties in the transition of care; and (d) scarcity of resources.

**(a)**  **Myths and misconceptions regarding PC**

Professionals acknowledged that there are several myths and misconceptions among patients, families, and the wider community regarding PC. The myth that PC hastens death, as well as the association of PC with terminality and abandonment, constitute barriers to timely referral. According to participants, it is necessary to invest in community awareness about PC, its benefits, and its person-centered approach.

*FG1: People often associate palliative care with people who are dying, so we sometimes face reservations about speaking openly as we may not be well interpreted (…). The lack of literacy in palliative care is very significant. Talking about death is still taboo.*
*FG2: People really associate palliative care with terminality and the idea that there is nothing more to offer. But after receiving PC, patients feel very good and appreciate this support.*
*FG3: Talking about palliative care is scary for many and there are many prejudices. We had a user who only accepted treatment here with us in the treatment room, and did not accept care at home (…). Families themselves also experience uncertainty and fear of dealing with losses… and this greatly affects adherence.*
*FG4: It is important to demystify the word “palliative” for the community in general. We recently had a case of a user who was an informed person, but who got it into his head that when he was referred to palliative care it was the end for him… he internalized that we were, in a way, giving up on him. Later, the family team went home after he was integrated into palliative care and he is now grateful because he is in his natural environment, in his home.*

**(b)**  **Early referral conditioned by the heterogeneity of disease trajectories**

The teams described the different moments in which they felt the need to refer more complex cases to specialized teams. In all FGs, it was evident that the lack of symptomatic control, a worsening functional state, and an increase in psycho-emotional and spiritual needs make it difficult for family teams to monitor the situation.

*FG1: Referral is necessary when we can no longer control the symptoms, when multiple decompensations or hospitalizations begin to occur. Certain types of therapeutic formulations are not available in PHC, for example, subcutaneous and transdermal or transmucosal medication.*
*FG2: When the pain is no longer controllable, or multiple side effects of the medication appear: constipation, dyspnea, lack of appetite and nausea…. We try to control it, but at a certain point we need help from specialized teams.*

Although participants describe the transition of care related to patient complexity, specific tools are not used for their assessment. At the same time, the difficulty in interpreting the assessment scales used by specialized PC teams was also mentioned.

*FG1: We know that there are tools that can be used to assess the complexity of patients, but they are also very extensive and not easy to apply (…). Sometimes we look at the palliative care team’s records and we see that they use a lot of scales, but then having to interpret or validate that assessment is sometimes difficult.*

On the other hand, the responsibility of professionals in the late transition to specialized PC was highlighted.

*FG5: We referred too late, we thought about it too late. Perhaps we don’t have the sensitivity to understand that it makes sense to refer right at that initial stage. And perhaps an initial assessment is more advantageous than an assessment “just to die”, so to speak.*

**(c)**  **Bureaucratization of processes and difficulties in the transition of care**

The bureaucracy associated with referral processes, the slow response times, and the limited response from the National Network of Integrated Continuing Care (RNCCI) constitute significant barriers for participants.

*FG3: On the computer platform, the social worker has to fill out one part, the nurse fills out another part, the family doctor fills out another part… All of this is a time-consuming process and the user is waiting, with the possibility of the request being denied…. The delay is a problem!*
*FG5: The referral form is terrible to fill out and our response capacity should be faster. There shouldn’t be so much bureaucracy, because patients and families are suffering…*

Professionals also criticize the lack of a user-friendly platform for referral to the RNCCI, which requires filling out a series of questions that, in most cases, are poorly adapted to reality.

*FG4: Very difficult and there are questions that don’t fit very well, details that are unnecessary, without relevance. Mandatory questions arise of a sexual nature that seem unnecessary to us, at least at the time of referral. It might make sense at a later stage, but not at that moment.*

These participants also expressed the lack of a proximity of care in a collaborative and integrated network where they could obtain advice and support in decision-making regarding patients with palliative needs.

*FG3: There are not enough community teams in PC to help us with the most complex cases. There is no proximity and this also makes it difficult to access answers.*
*FG4: We have the RNCCI, but we do not have a Community Palliative Care Support Team. We can only count on the in-hospital team, but we do not have access to them, only the hospital services can refer patients there.*

Another barrier to the safe transition of care results from the lack of coordination between primary and hospital health care, hindering the user’s integrated monitoring.

*FG1: Just think that when a person is hospitalized, they often lose contact with their family doctor, and communication between the two levels of care does not always exist.*

**(d)**  **Scarcity of resources**

The shortage of human resources in essential primary care to support palliative patients, such as psychologists and social workers, is a difficulty faced by participants.

*FG2: We lack resources, we realize the importance of psychosocial support and then, in practice, we lack the support of psychologists.*
*FG3: Social prescription here is still a myth, it is still a goal to be achieved. Obviously, it is through the social worker that the family often mobilizes home hygiene support, support for meals, transport and other benefits to which the user may be entitled. But the social worker is “general”, not just involved in the referral process or the palliative care team. It’s the only one that exists here, (…) and it ends up being overloaded.*
*FG4: We have very few resources. We have had a psychologist here at the Health Center for a short time now, but she is clearly not, in any way, sufficient for what we need. Therefore, either families have the possibility to appeal on their own, or unfortunately they are left without answers.*

The need to adequately understand the disease trajectories for early referral was also recognized. However, participants state that response capacity is largely determined by available resources.

*FG5: According to the person’s diagnosis, it is possible to anticipate their needs and we try to prevent the situation from evolving into symptomatic loss of control. We try to ensure that the person has the greatest comfort and the best care possible. However, we have a limited response capacity due to a lack of resources.*
*FG4: This is very unpredictable. It is obvious that there are situations, especially in cancer, when we can predict better, but others… it is very difficult to perceive when the person will start to decline. And so ideally we would have a faster response capacity. In other words, we risk referring people who didn’t exactly need it at that moment.*

#### 3.2.3. Theme 3–Ways to Mitigate Difficulties in Accessing and Referring to Specialized PC

The professionals suggested several strategies that could be used (and some that are already used) to mitigate difficulties in accessing and referring to specialized PC. In this context, the following subthemes emerged: (a) investment in PC training for PHC professionals; (b) customization of services to the needs of people with palliative needs; and (c) strengthening specialized community palliative care teams (CPCT).

**(a)**  **Investment in PC training for PHC professionals**

The disinvestment in PC in undergraduate training was recognized by all groups. Consequently, investing in training was considered a priority with an impact on both the quality of care offered and the referral process.

*FG2: It is very likely that things would be better if we had basic training in PC. Mandatory palliative care training should even be part of the General and Family Medicine internship. (…) It may even be necessary to decide whether it is time to make a referral or even to be more alert to the activities that we can or cannot do.*
*FG3: It is the general feeling that we do not have adequate training. In the case of younger professionals, only those who seek training at the master’s level are more up-to-date. Furthermore, mandatory training is insufficient or even non-existent (…) It is one of the areas in which we need ongoing training and updating.*
*FG4: The big issue here is that most of us don’t have PC training and we are on the front line! Until patients reach specialized teams, we are the ones who deal with them and their families. What we learn is based on acquired experience, and also on our sensitivity. Therefore, it would have been important to have some investment in the continued training of professionals.*

Professionals who have completed postgraduate studies in PC recognize gains in their clinical activity in terms of managing more complex cases and the need for referral to specialized units. The rest of the team also ends up benefiting from the presence of trained professionals, fostering numerous debriefing moments that allow discussion of cases.

*FG2: Training undoubtedly facilitates referencing itself. Those who have undergone training in the area are more sensitive to the problem and end up referring it much more, compared to colleagues who have not…*
*FG4: We have learned from those who are doing postgraduate studies within our unit. We ask a lot of questions and discuss the most problematic situations.*

**(b)**  **Customization of services to the needs of people with palliative needs**

Participants reported that it was necessary to dedicate more consultation time to meet the needs of palliative patients, since the time available in a “normal” consultation is not sufficient for monitoring the needs of patients and families.

*FG4: The truth is that a consultation time of 15 to 20 min is clearly not enough to deal with the complexity of the needs of the palliative patient and their family. Which means rescheduling after rescheduling, and families are left dissatisfied.*
*FG1: We fall short in terms of psychological and emotional support, largely due to the time constraints we face. It is necessary to manage the schedule to allow more consultation time to be reserved for these users.*

While some professionals can easily control the scheduling of appointments, others do not have this possibility. To solve this problem, participants mentioned the creation of a specific consultation in palliative care, as is already done for hypertensive, diabetic, pregnant patients, etc.

*FG1: Sometimes we don’t have control over appointment schedules. Therefore, only by creating a specific query in PC is it be possible to improve the quality of our current care provision.*
*FG3: The main problem is that we have to respond to the constant pressure we suffer from patients, family caregivers, coworkers and managers. We have to create priorities and there is not time for everything. The possibility of a PC consultation as part of the PHC service portfolio could be a solution similar to what we do in other areas. However, this implies the secure allocation of human resources.*

The need for an integrative care model was highlighted as an ideal model of action in which early referral to palliative care is combined with primary health care. The possibility of PHC teams including professionals with training in PC would facilitate intervention with palliative patients, as well as serve as a bridge between PHC services and specialized teams in PC.

*FG4: Basic palliative care units should be created in primary health care. Just as there are additional portfolios for alcoholism, smoking cessation, obesity, etc., there should be a basic palliative consultation by trained colleagues to help manage symptoms, provide psychological first aid, etc. It would be an easy and relatively cheap way for the Unified Health System to democratize access to palliative care for a large part of the population that is currently unable to benefit from it.*
*FG3: Create teams… for example, in each LHU a team should be created to carry out specific palliative consultations. This was envisaged a long time ago, but it never happened.*
*FG2: Having the possibility of shared monitoring makes perfect sense. (…). We don’t have the time that community palliative care support teams offer, they have visits lasting two hours… We don’t have that time, we can’t provide that support. Therefore, we need an integrated approach and greater proximity between generalist care and specialized PC care.*

**(c)**  **Strengthening specialized community palliative care teams (CPCT)**

In all FGs, participants reported added value in strengthening CPCTs, as they support decision-making and provide consultancy when necessary.

*FG1: I have already had conversations with colleagues at CPCTs about pain control therapy, using co-analgesics that we do not usually use. With these teams, we don’t feel helpless.*
*FG2: They have the team’s cell phone, to provide support, both for patients and for us, colleagues. Fortunately, we have the community team and a good relationship with them. We are a privileged unit and I think this has a lot of influence on our performance.*

Another positive aspect described by participants is the speed of the CPCT response. Although CPCTs have limitations in offering home care to all palliative patients, they offer other services such as consultancy, telephone support, and teleconsultation.

*FG5: They are quick to respond, often responding within the same week. Sometimes they suggest a first joint visit with the family doctor, who in this case is the person who already knows the patient and who proposes the referral. Even if they are not able to make a home visit, there are other forms of support such as telephone assistance and teleconsultation.*

## 4. Discussion

Family doctors and nurses play a central role in accessing and referring patients to PC, since the response provided by specialized palliative care does not meet the needs of the population [12]. The skills of these professionals include approaching the patient’s suffering, basic symptom management, and developing a treatment plan aligned with the person’s preferences, thus respecting their autonomy [51,52]. Although participants described relative ease in identifying and assessing palliative needs, the majority revealed ambivalence in the process of assessing non-oncological palliative needs, with difficulty in identifying the needs of these patients and the apparent devaluation of situations associated with aging and long disease trajectories. In fact, according to the available evidence, frail older people, people with non-malignant diseases, and people from ethnic minorities have unequal access to PC. Given that they tend to access these services later and with less functional reserve, many are never identified as having palliative needs [33,53,54]. PHC professionals developed a person-centered approach, which has demonstrated positive effects on health outcomes, including better patient–professional relationships and higher patient satisfaction rates [55]. According to the literature, family doctors and nurses are essential for providing EoL care, due to the strong, close relationship built over time, which allows for detailed knowledge of the patient and family’s history [8]. Although participants consider themselves to be in a privileged position of proximity to users (which facilitates communication), they admit to difficulties in communicating bad news and the need for training in this area [56].

The findings highlighted several difficulties in accessing and referring to specialized PC. Emphasis was placed on myths and misconceptions regarding PC, so it is essential to invest in PC literacy among patients, caregivers, and the wider community [57,58,59,60]. Although professionals acknowledge the increased complexity of patients and lack of symptom control as the main reasons for referral to specialized PC, they recognize that these are often late. According to previous studies, this fact may be related to difficulties in estimating prognosis, disease trajectory, and timely recognition of the end of life [19,30,32,33,59]. Other barriers include the scarcity of resources, bureaucratization of referral processes, and difficulties in communication between health professionals, conditioning the fluidity in the articulation of care [7,61].

Investment in training was identified by participants as an improvement strategy with an impact on the care they offered and also on referral processes. The education and training of health professionals in PC have been reported as inadequate in several studies carried out in different populations, particularly with regard to the complexity of care and the diagnosis of palliative needs [30,61]. To improve professionals’ skills, it is necessary to provide learning opportunities and educational resources [8]. Customizing services for people with palliative needs was also a strategy proposed by participants, as a way of overcoming the lack of consultation time at PHCs to meet the needs of these patients in the best and most complete way possible [57,62]. These strategies involve adapting the medical or nursing schedule to increase the consultation time allocated to these patients or implementing a specific consultation model for this type of patient [63]. It was also suggested that a consultation be implemented by primary health care teams with specialized training in palliative care. This could serve as a bridge between family doctors and nurses and specialized hospital care. The possibility of consultancy, the use of effective communication channels, collaborative work, and the existence of an integrated care network are tools that increase the responses of health professionals [64,65]. In the present study, participants recognized the importance of CPCT as teams that meet these criteria, given their collaborative work model and proximity to the community [66]. Furthermore, participants highlighted the need to increase the number of these teams, which provide more rapid responses, as an improvement strategy to be implemented.

### 4.1. Strengths and Limitations

The present study presents a set of strengths and limitations. Given the lack of research in Portugal on this topic, this study contributes to the expansion of available knowledge regarding the experiences of PHC professionals. This study highlights the role of family doctors and nurses as key elements in ensuring global access to PC, both due to their fundamental role in providing PC and their importance in referrals to specialized teams. Furthermore, the integration of the subjective perspectives of professionals provides a comprehensive view of the reality of PC in Portugal. In methodological terms, the researchers rigorously followed the different phases of the research. To ensure the credibility of the study, the researchers maintained reflexivity throughout the research process, and each research phase was discussed and documented in detail. In parallel, the sample provided variations in the experiments and attained data saturation.

Regarding the limitations of the study, the fact that the participants within each FG knew each other could have influenced their interactions. FGs can exhibit subject bias, given that dominant participants or the moderator can influence individual and group opinions, which may result in the non-expression of some individual perceptions and experiences. To mitigate the risk of social desirability bias, data collectors used techniques to establish rapport with participants, such as using probing questions, requesting stories and examples, and providing assurances. While focus groups can be valuable for exploring specific topics and understanding group dynamics, relying solely on them without methodological triangulation can lead to a less comprehensive and potentially biased understanding of the research question. Further mixed methods may identify convergent or divergent patterns of phenomena. Although using a qualitative vignette is an effective projecting strategy, this can elicit answers from participants that they prefer to withhold. To address this claim, participants were assured they were not required to connect the scenario to their personal experiences unless they chose to do so. Although the present study involved three different local health units in the central region of the country, we admit that the practices of PHC and PC professionals, as well as the organization of these services, vary with geographic context, limiting the transferability of the findings.

### 4.2. Implications for Practice

This study offers a deeper understanding of the Portuguese reality in terms of the articulation between primary health care and PC. It identified the main challenges faced by PHC health professionals when providing PC and in the transition of care, highlighting strategies that they apply or recommend for improving clinical practice. In Portugal, PC education in undergraduate programs, particularly within the nursing and medical fields, is being developed but still faces challenges in terms of curriculum integration and practical experience. While there is growing awareness of the need for PC education due to an increased prevalence of chronic diseases, there is a gap between theoretical knowledge and practical application that reinforces the need for better curricula integration of PC. This study also highlighted the importance of training and continuous professional development in PC for PHC providers. Improving education and support for PHC teams may enable them to be more proactively involved in identifying patients with palliative needs and initiating early referral. The stereotypes that exist in the community regarding PC make it urgent to invest in health literacy, in order to highlight the work carried out by PC services [67,68]. Furthermore, research highlights the need to strengthen communication and collaboration among healthcare professionals. Fragmentation and lack of integration are often expressed as barriers, suggesting the need for more structured interprofessional collaboration and shared care models [69,70]. The scarcity of resources and the bureaucratization of processes are important issues on the political agenda, and it is necessary to invest in financing PHC and simplifying referral processes.

## 5. Conclusions

To the best of our knowledge, so far, no other studies have analyzed the practices, access, and referral to PC from the perspective of PHC professionals. The findings were organized into three core themes: (1) the contours of palliative action developed by PHC teams; (2) barriers to access and safe transition between PHC and specialized PC; and (3) ways to mitigate difficulties in accessing and referring to specialized PC. This study highlights the fundamental role of PHC professionals in providing primary PC, identifying PC needs, and referring patients to PC early, while exposing the systemic and interpersonal challenges that hinder these processes. To overcome these challenges, it is essential to invest in the development of integrated care models that promote practical, low-bureaucratic referral processes and capture the human resources necessary for adequate follow-up of users. Measures should strengthen collaboration between PHC and PC services, as well as promote the improvement of professional training, particularly in communication skills. Health education must be promoted to demystify PC among the population. The findings also highlight the importance of CPCTs in PC as fundamental agents to improve access and support PHC professionals in care decisions and practices. By recognizing and acting on these implications, health systems can move toward a more equitable, timely, and person-centered PC, anchored in strong primary health care and supported by informed and empowered communities.

## Figures and Tables

**Table 1:** Participants’ background (N = 34).

			FG1	FG2	FG3	FG4	FG5
**Participants number**			6	7	7	6	8
**Age** (Mean ± SD)			35.8 (7.0)	42.9 (13.4)	50.9 (10.6)	44.5 (8.9)	46.2 (5.3)
**Sex**	Female		5	6	6	5	4
	Male		1	1	1	1	4
**Profession**	Family Doctor	Specialist	3	5	4	2	5
		Resident	3	2	0	0	0
	Nurse	Specialized Family Nurse	0	0	0	2	3
		Without Specialty	0	0	3	2	0
**Years of professional experience** (Mean ± SD)	In health care, globally		9.5 (7.8)	17.1 (15.9)	26 (11.3)	21.3 (11.2)	19.6 (6.3)
	In Primary Care		8.2 (7.6)	15.7 (15.5)	20.4 (11.2)	13.2 (4.2)	13.8 (6.9)
**Training in PC**	No		2	6	5	4	6
	Yes		4	1	2	2	2
**Perceived competence level for PC** on a scale from 0 to 10 (Mean ± SD)			3.5 (1.2)	2.9 (1.5)	4.3 (1.1)	5.3 (1.9)	5.8 (2.4)

FG: focus group; PC: palliative care; SD: standard deviation.

**Table 2:** An overview of the themes and subthemes.

Themes	Subthemes
Theme 1—The contours of palliative action developed by PHC teams	(a) Identification of palliative needs (b) The primacy of the therapeutic relationship (c) Limitations in response capacity
Theme 2—Barriers to access and safe transition between PHC and specialized PC	(a) Myths and misconceptions regarding PC (b) Early referral conditioned by the heterogeneity of disease trajectories (c) Bureaucratization of processes and difficulties in the transition of care (d) Scarcity of resources.
Theme 3. Ways to mitigate difficulties in accessing and referring to specialized PC	(a) Investment in PC training for PHC professionals (b) Customization of services to the needs of people with palliative needs (c) Strengthening specialized community palliative care teams.

## Data Availability

All data generated or analyzed during this study are included in this article. This article is based on the first author’s Master’s dissertation in palliative care at the School of Health Sciences—Polytechnic University of Leiria (supervised by Carlos Laranjeira).

## Supplementary Materials

The following supporting information can be downloaded at: https://www.mdpi.com/article/10.3390/healthcare13131576/s1. Supplementary Table S1: Consolidated criteria for reporting qualitative studies (COREQ): 32-item checklist. The reference (Tong et al., 2007 [41]) is cited within the Supplementary Materials.

## Abbreviations

COPD	Chronic Obstructive Pulmonary Disease
FG	Focus Group
LHU	Local Health Unit
PHC	Primary Health Care
PC	Palliative Care
CPCT	Community PC Teams
RNCCI	National Network of Integrated Continuing Care (Rede Nacional de Cuidados Continuados Integrados *in Portuguese*)
